# Knowledge, attitudes, ethical and social perspectives towards fecal microbiota transplantation (FMT) among Jordanian healthcare providers

**DOI:** 10.1186/s12910-021-00587-6

**Published:** 2021-02-27

**Authors:** Amal G. Al-Bakri, Amal A. Akour, Wael K. Al-Delaimy

**Affiliations:** 1 School of Pharmacy, Department of Pharmaceutics and Pharmaceutical Technology, The University of Jordan, Amman, 11942 Jordan; 2 School of Pharmacy, Department of Biopharmaceutics and Clinical Pharmacy, The University of Jordan, Amman, 11942 Jordan; 3 Department of Family Medicine and Public Health, University of California San Diego-School of Medicine, La Jolla, USA

**Keywords:** Fecal microbiota transplant, Social and religious perspectives, Attitudes, Ethics, Jordan

## Abstract

**Background:**

Fecal microbiota transplant (FMT) is a treatment modality that involves the introduction of stool from a healthy pre-screened donor into the gastrointestinal tract of a patient. It exerts its therapeutic effects by remodeling the gut microbiota and treating microbial dysbiosis-imbalance. FMT is not regulated in Jordan, and regulatory effort for FMT therapy in Jordan, an Islamic conservative country, might be faced with unique cultural, social, religious, and ethical challenges. We aimed to assess knowledge, attitudes, and perceptions of ethical and social issues of FMT use among Jordanian healthcare professionals.

**Methods:**

An observational, cross-sectional study design was used to assess knowledge, attitudes, and perceptions of ethical and social issues of FMT among 300 Jordanian healthcare professionals.

**Results:**

A large proportion (39 %) thought that the safety and efficacy of this technique are limited and 29.3 % thought there is no evidence to support its use. Almost all (95 %) responded that they would only perform it in certain cases, if ethically justified, and 48.3 % would use it due to treatment failure of other approaches. When reporting about reasons for not using it, 40 % reported that they would not perform it due to concerns about medical litigation, fear of infections (38 %), and lack of knowledge of long safety and efficacy (31.3 %). Interestingly, all practitioners said they would perform this procedure through the lower rather than upper gastrointestinal tract modality and the majority will protect the patient’s confidentiality via double-blinding (43.3 %). For a subset of participants (n = 100), the cultural constraints that might affect the choice of performing FMT were mainly due to donor’s religion, followed by dietary intake, and alcohol consumption.

**Conclusions:**

Our healthcare practitioners are generally reluctant to use the FMT modality due to religious and ethical reasons but would consider it if there was a failure of other treatment and after taking into consideration many legislative, social, ethical and practice-based challenges including safety, efficacy and absence of guidelines.

## Introduction

Fecal microbiota transplantation (FMT) is a procedure involving the transfer of stool from a healthy screened donor into the intestinal tract of a diseased recipient. FMT is claimed to possess a therapeutic effect by remodeling the gut microbiota and treating microbial dysbiosis, which is often defined as an “imbalance” in the gut microbial community that is associated with disease [1–4]. Traditionally, FMT is prepared as a crude fecal matter using a manual method where the “fecal matter, or stool, is collected from a tested donor, mixed with a saline or other solution, strained, and placed in a patient, by colonoscopy, endoscopy, sigmoidoscopy, or enema” [5]. Recently, a standardized automated washed microbiota transplant (WMT) preparation method was introduced and was found to significantly reduce FMT-related adverse events.

FMT has been used to successfully treat recurrent *Clostridium difficile* infection [6, 7] and guidelines towards its safe use are continuously evolving. As such, the Infectious Diseases Society of America (IDSA) [8], Society for Healthcare Epidemiology of America (SHEA) [9], and The World Society of Emergency Surgery (WSES) [10] have recently updated the Clinical Practice Guidelines for *Clostridium difficile* Infection (CDI). The updated ISDA and SHEA guidelines include the use of FMT as a CDI treatment in the second or subsequent recurrence with strong to moderate strength of recommendation and quality of evidence [8, 9]. According to the Food and Drug Administration (FDA), regulation of the use of FMT for recurrent CDI should clearly be explained as being an experimental approach. As such, the use of FMT is a mix of a clinical trial and standard care [11].

The long history of FMT has witnessed an evolution in the methodology, clinical strategies, and delivery methods [12]. Therefore, modernized FMT guidelines have been formulated [6, 13, 14]. Nevertheless, efforts are still needed for establishing standardized protocols for stool preparation, FMT administration and delivery methods, donors and recipients selection criteria [15], and stool banking [16, 17]. Meanwhile, there has been great interest in FMT applications and growing evidence suggests its potential use in the management of GI conditions other than recurrent CDI [18], including ulcerative colitis [19], cardiometabolic syndrome [20], Crohn’s disease [21], irritable bowel syndrome [22], and some neurological disorders such as multiple sclerosis [23] and Parkinson’s disease [24]. Nevertheless, these potential therapeutic uses face many challenges [25], and thus more safety and efficacy studies are still needed. Moreover, technical, legislative, regulatory, ethical and social concerns in creating a standardized treatment modality should be addressed and resolved.

FMT therapy had faced and is still facing numerous regulatory, ethical, cultural, and social challenges. Ethical challenges include [26], (1) ”informed consent and the vulnerability of patients”; (2)” determining what is a suitable healthy donor”; (3) “safety and risk”; (4) “commercialization and potential exploitation of vulnerable patients”; and (5) “public health implications” [26]. Personal identity and family relations [27–29] have been identified as additional ethical challenges. The findings that altered microbiota can be passed to the offspring [30] and the possibility of family members to be potential secondary recipients, raised calls for the consideration of the ethical complexity and challenges associated with microbiome research in FMT procedures and regulations [31]. Moreover, due to the cultural and religious constraints of certain types of diet and the effect of dietary intake on individual's microbiome composition, dietary intake might be considered an ethical challenge in FMT consenting procedure [28–31]. All these challenges make it very difficult to demarcate the regulatory framework. Indeed, the regulatory status of FMT has been changed several times and is continuously modified [32, 33].

Despite the reported therapeutic effects of FMT in recurrent CDI management, its use is limited by many factors, including lack of specialized centers, difficulties with donor selection and recruitment, and difficulties related to regulation and safety monitoring [17, 34], in addition to the social and ethical challenges described above. In contrast to the long standing FMT use in China, FMT is not regulated as a therapeutic tool in Jordan nor is officially practiced. In light of the growing evidence of FMT therapeutic effectiveness in the management of different GIT dysbiosis related disorders, we expect it will become an approach used by Jordanian practitioners in the future. Nevertheless, the differences in the cultural, social, and religious make up of Jordanian and Islamic conservative tradition compared to China or Western countries might entail unique ethical challenges towards FMT therapy and thus specific regulations for its use. Indeed, it has been shown that the cross-cultural differences between Chinese [26] and Western cultures impacted the shaping of their FMT regulations [35, 36].

The aim of our current study was to investigate the knowledge, attitudes, and perceptions of ethical and social issues regarding FMT uses by Jordanian healthcare providers to highlight the ethical challenges in the context of Jordan’s cultural and social makeup.

## Methods

### Study design, settings, and subjects

This was an observational, cross-sectional study design, the aim of which was to assess knowledge, attitudes, and perceptions of ethical and social issues of FMT among Jordanian healthcare professionals. The study was conducted in Amman, Jordan between June and August 2019. Using convenient sampling, 300 various healthcare practitioners, including gastroenterologist and/or internists, medical doctors, nurses, medical laboratory technicians, and pharmacists were invited to participate in the study and asked to fill a paper-based questionnaire. The goals of the study, as well as the questionnaire, were thoroughly explained to each participant before getting their verbal consent to participate. Their participation was voluntary and their responses were anonymous. This study was approved by the Institutional Review Board of The Jordan University Hospital (IRB no. 80/20/9/535) dated 3/11/2019.

### Questionnaire development

The questionnaire was based on that used by a previous study by Ma et al. [26] with some modifications. The latter is distributed under the terms of the Creative Commons Attribution 4.0. International License (http://creativecommons.org/licenses/by/4.0/).

In brief, the questionnaire consisted of four sections comprising 20 items: general knowledge and attitudes towards FMT (four items); perception of ethical concerns (nine items); belief about social and regulatory issues (four items); and views about FMT bank ethics (three items). Question formats included single choice, multiple-choice, and written short answer. To this questionnaire, we added questions about cultural constraints including religion, dietary intake and alcohol consumption, for a subset of participants (n = 100). Moreover, using an open-ended question, participants were asked to write any other comments regarding FMT that they wish to make.

### Sample size calculation

For the questionnaire, sample size was calculated based on O’Rourke et al. [37], where it is recommended that the number of subjects should be 5–10 times the number of items, or 100. Given that we have 21 items in our questionnaire, a sample size of 105–210 participants was considered representative for the purpose of this study.

### Statistical analysis

Data were analyzed using Statistical Package for Social Science (SPSS®) version 22 (SPSS® Inc., Chicago, IL, USA). The descriptive analysis was done using frequencies and percentages. Chi-square (or Fisher’s) test was used to compare practitioners who were familiar and/or involved with FMT vs. those who were not. An arbitrary negative score was created from the negative views about FMT, assigning a value of 1 for answers with a negative attitude and 0 for positive attitudes. Independent student’s t-test was used to compare the score between practitioners familiar with FMT vs. those who are not. In addition, ANOVA test was done to check the difference by profession. P-value less than 0.05 was considered statistically significant.

## Results

Data were collected from 300 healthcare professionals. Table 1 below describes results as frequency (n) and percentage (%). Most of the participants were gastroenterologist (38 %) followed by medical doctors (23.7 %). The vast majority (95.7 %) did not perform FMT but have heard about it.

### Ethics

Regarding ethical issues, it seems most of the responders were skeptical and not supportive of using the FMT method. A large percent (39 %) thought that the safety and efficacy of this technique are limited and another 29.3 % reported that there is no evidence to support its use. When asked if the methods were medically indicated and ethically approved would they use it, still only 5 % would refer a patient for FMT. About 40 % would not perform it due to concerns about medical litigation, followed by fear of infections (38 %), and lack of knowledge of long-term safety and efficacy (31.3 %). But 48.3 % would do it when other treatments fail and another 29.7 % would do it if there was a need for organic or natural treatments.

The majority will protect the patient’s confidentiality via double-blinding (43.3 %). Not everyone was willing to inform the patients about all risks as some would inform them about actual physical risk from the procedure and others will inform patients depending on their comprehension. Concerning the FMT bank, all participants viewed that there is a problem in donor’s anonymity and data de-identification, and 47.7 % were worried about the consent methods. The ethical concerns were numerous and included the mode of informed consent, privacy protection, and ownership of samples.

### Perceptions about the use and efficacy Of FMT

Only 20.7 % believed that FMT was overrated, and 42% did not agree that FMT value is overrated, and the rest did not know. Interestingly, all practitioners would perform this procedure through the lower gastrointestinal rather than the upper gastrointestinal tract. A total of 43% supported the statement that FMT negatively impacts the patient’s dignity. As for social and regulatory issues, 87% believed that the application of FMT should be suspended and it is not urgent to apply, 84 % believe that FMT will not have other future applications, and 100% said that it should not be used as the first line for CDI. Barriers to the use of FMT were due to lack of guidelines (40.3%), and unknown mechanisms of action of this treatment (33.7%). “Do-It-Yourself” (DIY)-FMT—meaning lay individuals adopting FMT clinical techniques performed on and/or by themselves at home. Social media has facilitated widespread exposure to and awareness of the relationship between the gut microbiome and human health [38]. Given the availability of the necessary raw materials, the straightforward technique of FMT administration, and anecdotal success stories online, numerous websites have already sprung up advertising home DIY-FMT kits as a direct-to-consumer (DTC) product. The concept of commercialization of FMT raises concerns regarding proprietary rights, accessibility of data and biological material, and the implications of DTC products [39]. With regards to commercialization, 81.3 % of participants thought that DIY and DTC advertisement is common in other areas. Internationally, DIY practices have been developed to include different therapeutic areas such as DIY-FMT [38], DIY-Diet [40], DIY-Automated Insulin Delivery [41]. In Jordan people are becoming increasingly familiar with drugs and their brand names and are practicing self-medication [42] including the use of antimicrobials [43], which can be considered as a concerning DIY health practice area.

### Cultural aspects

For a subset of participants (n = 100), we asked about the cultural constraints that might affect the choice of performing FMT, for 52 % it was the religion, then dietary intake (25 %), and alcohol consumption (23 %). Due to the scarce number of practitioners who fall in the category of being familiar and/or involved in FMT (13 out 300), the comparison of different variables according to this parameter would not be accurate due to the large difference in sample size between these categories. However, some results were worth mentioning. Those who were familiar with FMT were gastroenterologists and/or internists and only one of them did not think that it is a promising modality. All of them (n = 13) would not recommend FMT due to concerns about infection, while 36.2 % (n = 104) of those who are not familiar with FMT have such concerns (p-value = 0.003). Moreover, half of those who are familiar would inform patients about physical risks vs. 16.7 % in those who are not familiar with FMT informing patients about physical risks (p < 0.002). Alcohol was the main cultural concern among those who did not perform FMT (25.3 %), and dietary intake was the concern of those who did perform FMT (44.4 % vs. 23.1 %), and religion was equally concerning for both groups.

**Table 1:** Questionnaire items 1–20. Results are represented as frequency (n) and percentage (%)

Parameter	N	%
*General*		
*Profession* Gastroenterologist and/or internist Medical doctor/ Nurse Medical Laboratory technician Pharmacist	114 71 34 53 28	38.0 23.7 11.3 17.7 9.3
*Familiarity with FMT* Yes, I have performed FMT No, I am not familiar but I heard of it No, I am not familiar nor heard of it	13 287 0	4.3 95.7 0
*Ethical issues*		
What do you think of FMT?* A promising treatment modality for some diseases The efficacy and safety of FMT is very limited The current data is not sufficient to support the use of FMT I do not know/I am not quite sure	18 147 88 19	6 39 29.3 6.3
*If it is medically indicated and ethically approved, would you refer FMT to patient?* Yes It depends (e.g. conventional treatment failure) No	15 285 0	5 95 0
What is/are the reason/s for you to recommend FMT and which you will also inform patients?* Clinical efficacy Safety Failure of conventional treatment More “natural” and “organic” Avoidance of antibiotics Others	27 57 145 89 19 81	9 19 48.3 29.7 6.3 27
*What’s the reasons for you not to recommend FMT and which you will also inform patients?* Unproven treatment and unknown mechanism Infections Long-term risk and safety unknown High expectation from patients puts pressure on physicians Not a standard treatment, easily cause medical litigation Others	81 114 94 18 121 4	27 38 31.3 6 40.3 1.3
*Is FMT overrated (efficacy exaggerated and risk downplayed)?* Agree I do not agree I do not know	62 126 112	20.7 42.0 37.3
*Would you inform patients about possible risks?* I would inform about all risks I would inform about actual physical risk It depends on the comprehensive ability of the patient	132 55 113	44.0 18.3 37.7
*Do you think FMT has negative effect on patient’s dignity?* Agree Disagree I do not know	129 75 96	43.0 25.0 32.0
*What do you think is the optimal modality to deliver FMT?* Lower Upper	300 0	100 0
How to protect privacy/confidentiality?* Both donor and recipient are double-blind Donor should be anonymized Establish standardized fecal microbiota bank FMT patients should have private ward or room Ensure confidentiality of patient information during communication with others	130 41 27 89 83	43.3 13.7 9 29.7 27.7
*Cultural constrains (only for 100 participants)* Religion Dietary intake Alcohol consumption	52 25 23	52 25 23
*Commercialization*		
*What do you think of DIY*^1^ *and DTC*^2^ *of FMT* Worrying It is common in other areas No concerns	55 244 1	18.3 81.3 0.3
*Future application (e.g. skin care)* Likely (with concerns) Unlikely Fictional	48 252 0	16.0 84.0 0
*Social and regulatory issues of FMT*		
*Do you think it is urgent to apply FMT?* Yes urgent It should be suspended	39 261	13.0 87.0
*Charging standard for FMT?* Yes, ASAP No need	41 259	13.7 86.3
*FMT as first-line for CDI?* Yes No	0 300	0 100
Barriers to FMT promotion?* Unknown mechanism The yuck factor and anesthetic challenges Stained doctor-patient relationship Lack of pharmaceutical investment No official guidelines and regulations	101 141 23 25 121	33.7 13.7 7.7 8.3 40.3
*Fecal microbiota bank*		
Ethical aspect(s) concerns* Informed consent mode Privacy protection of personal information De-identification and anonymity of donors Ownership and property of samples Access regulation to data and sample Future use of specimen and re-contact	143 113 2 103 57 54	47.7 37.7 0.7 34.3 19 18
*Justice in allocation of benefits and burden* Patients should receive benefits Patients should not I do not care	117 26 157	39 47 52.3

*1 DIY* Do-It-Yourself, *2 DTC* Direct-to-Consumer

*Questions were “choose all what apply” so percentages add up to more than 100%

Almost all the healthcare providers have heard of FMT, but only 4.3 % performed or were involved with this procedure. An independent t-test showed that there was no significant difference in the negative views’ score between practitioners familiar with FMT (n = 13; mean = 9.9, SD = 2.1) compared to those who are not (n = 287; mean = 10.3, SD = 2.1); (p = 0.55). Although medical doctors had higher negative scores than other healthcare practitioners, there were no statistically significant differences between scores them (p = 0.15). The mean negative views’ score regarding FMT among different healthcare practitioners are shown in Table 2.

**Table 2:** Mean negative views’ score regarding FMT among different healthcare practitioners

Profession	N	Mean	SD*
Gastroenterologist and/or internist	114	10.4	1.9
Medical doctor	71	10.8	1.8
Nurse	34	10.0	1.5
Medical laboratory technician	53	9.76	1.5
Pharmacist	28	9.79	1.4
Total	300	10.2	1.8

*Standard Deviation

In addition, an open-ended question allowed participants to express their views about FMT in a category called “others”. Seventy of them answered this question, 17 % (n = 12) of them expressed religious objections and 30 % (n = 21) of the participants declared the need to consider the religious point of view and to seek Fatwa. Moreover, 41 % of them were concerned about lack of experience and clinical trials in the Arab region (n = 29), and 11 % (n = 8) thought it should be sought as last resort, with strict monitoring, or might have role in future.

## Discussion

FMT harmonized regulations are lacking and the current regulatory status ranges from non-existing to strictly regulated [32]. For now, the US FDA had classified FMT as a live biotherapeutic drug that requires the submission of an Investigational New Drug application for its therapeutic uses [33]. CDI has been recently exempted from IND application filing, which was a decision that was received with high appreciation by clinicians to use FMT in a fatal ailment. Meanwhile, strict regulation and control over the use of such treatment were recommended by, Renzong Qiu, 2017 [44]. Although, FMT has received great attention lately, there is still a gap in the understanding of FMT around the world, even in countries where it is being used [45, 46]. A wider acceptance of this therapy can be achieved by the implementation of regulations addressing the ethical and social issues facing its application such as the autonomy and the privacy of patients and donors, promoting research investigating its safety and efficacy, and the use of standardized methods in its preparation and application including stool banking [12, 16, 17]. Moreover, to promote its dissemination to countries in the Middle East,  such as Jordan, country specific social norms, tradition, customs and religious backgrounds, and structures should be taken into consideration towards introducing and regulating FMT [44].

Our results demonstrated that the majority of the respondents heard of FMT treatment but did not practice it. In contrast to Jordan, where FMT is not regulated nor practiced yet, FMT has been practiced in China since the fourth century where traditional Chinese medicine used yellow soup, fecal slurry, orally to treat food poisoning and diarrhea [26, 47]. This justifies the high familiarity of this treatment modality among Chinese clinicians [26]. Nevertheless, the familiarity does not guarantee experience in using it by clinicians; Zipursky, et al. [48] in their study reported that physicians have limited experience with FMT despite having treated patients with multiple recurrent CDIs.

In general, our study population was not enthusiastic about nor supportive of the introduction of such treatment. They did not see its promising utility for other future applications. Barriers towards the promotion and recommendation of FMT include mostly the absence of official guidelines and regulations followed by the risk of infections and long-term risk and safety. This is in concordance with Kelly et al. [49] who reported on physicians’ attitudes towards FMT in 2010 at the American College of Gastroenterologist meeting. They found that 40 % of physicians who had heard of FMT were not willing to try it, pending further demonstration of its efficacy safety. Nevertheless, Kelly et al., showed that physicians’ recommendation was positively influenced by patients’ perceived acceptance [49, 50]. This was not what our respondents think. In general, unwillingness for recommending FMT treatment were related to many factors; the limited knowledge among the study population [51], the limited practicing numbers [36], and the “yuck”factor [52]. Other reasons for physicians not offering or referring a patient for FMT were; “not having the right clinical situation’, “the belief that patients would find it too unappealing”, and “institutional or logistical barriers” [48]. In his commentary, Brandt et al., [52] related physicians’ hesitation to recommend FMT to the limited randomized controlled trials to show effectiveness and safety. He predicted that patients’ needs in addition to the availability of aesthetically acceptable formulations are influential parameters towards the acceptance of this treatment modality among physicians. Indeed, we found that the lower part of GI was the only acceptable route of administration of FMT. This might affect how the accepted FMT formulation will need to be regulated in Jordan in the future.

In support of the international legislations, our respondents will not recommend FMT as a first-line treatment, but only recommend it when there is a failure of conventional treatment or they want organic natural treatments. This is in agreement with the Iranian clinicians and gastroenterologists’ attitudes who reported a willingness of accepting FMT as a therapeutic option if it is scientifically justified and ethically approved given it was used as synthetic microbiota rather than FM [53].

Clinical efficacy is a crucial factor that maintains patients’ positive attitudes towards fecal microbiota transplantation [54] and physicians advising and referring patients to FMT treatment modality. The reported physicians’ responses regarding the efficacy and safety of FMT were diverse. While a major concern about FMT efficacy and safety was reported among Chinese clinicians [39], Zipursky et al. [48] have reported minor doubts about FMT’s efficacy and safety among physician respondents at Dartmouth-Hitchcock Medical Center and Baylor College of Medicine (Texas, USA).

In light of the above-described barriers and limited efforts in increasing the awareness of the uses and efficacy and safety of FMT treatment modality, we predict that the introduction and the regulation of this treatment modality in Jordan is not going to be soon. Accordingly, efforts should be put forth for increasing awareness about its utility and effectiveness and to highlight the ethical and cultural/religious challenges towards its application such as patients’ vulnerability, donor’s anonymity and data de-identification and the consenting procedure. Moreover, legislative and ethical challenges facing the establishment of biobanks in Jordan including privacy and confidentiality, specimen ownership and informed consent should be addressed [55]. According to the US FDA, during the investigational use of FMT, the potential risks and benefits including the unknown risks and the long-term risks should be clarified for qualified patients during the consenting procedure (Food and Drug Administration 2013). Consenting is an ethical challenge in FMT, which was recognized by close to 50 % of our participants. The FMT consenting procedure should consider patients’ vulnerability, unforeseen long term risks, and limited knowledge of the actual benefits and risks to the treatment in addition to the universal ethical requirement of biomedical research [56]. Ma et al. [26] believe patients’ compromised decision-making capacity and vulnerability are the main challenges to informed consent. They consider CDI patients vulnerable, and desperate individuals who can be easily affected by emotive language as being natural and safe whether from physicians or the media. This was opposed by Bunnik et al. [11] who believe that it is not the vulnerability or capacity to consent but rather the inadequate information that poses difficulties with regards to the FMT consenting procedure.

Other important challenging parameters in the consenting process are cultural/religious or personal/ideological food restrictions of stranger donor. In their commentary [57], authors questioned whether informed consent to FMT can be obtained without information about the donor’s diet. This an important ethical challenge that is very relevant to our region’s population that is mostly Muslim thus observing the religious commitment to halal nonalcoholic containing foods and beverages is essential. Our respondents think that religion, dietary, and alcoholic consumption will be considered as a barrier in patient’s acceptance of FMT. Accordingly, we perceive that it could be necessary to declare the donor’s dietary habits to obtain an autonomous decision in this region.

An important parameter that was highlighted by the respondent’s comments was the need to consider the religious point of view and to seek Fatwa. This was declared by 30 % of the participants in addition to their perception of the need for more knowledge about safety. Therefore, we concluded that our healthcare practitioners are reluctant to use FMT because of concerns about safety and religious beliefs. Ma et al. [58] highlighted important cultural and religious beliefs that might affect the public acceptance of FMT. Some people might consider FMT an unsanitary treatment, and some will limit the donor to those who eat specific food, such as vegans, or those with a specific religion such as Muslim patients who might not accept fecal transplant from non-Muslim donors. All these barriers entail the importance of demarcating region-specific FMT regulations that take into consideration the cultural and religious background of the public.

Although, there is a growing awareness of ethics in human research, nevertheless Alahmad et al. have shown that research ethics regulations and guidelines in Middle Eastern Arab countries suffer from various degrees of deficiencies with regards to ethical protection [59]. They recommended that social norms, traditions, customs, and familial ties should all be taken into consideration when developing policies and regulations. In interviews with medical professionals from the Middle East Alahmad et al. [60] reported the social importance of protecting confidentiality, de-identification, and anonymity of donors scored 100 % as being an ethical concern in conducting FMT among the Jordanian clinicians. They mostly agreed that confidentiality can be protected by double blinding both the donor and the receiver and to ensure the confidentiality of patient information during communication with others.

### Limitations

Firstly, our study adopted convenient sampling from the capital of Jordan (Amman), therefore the findings may not be generalizable to other provinces or worldwide. However, the objective of this study was to assess the perceptions of healthcare providers, regarding ethical and social concerns about FMT, as the first such study among this population and we do not expect our results will substantially change among other Jordanian physicians. Secondly, we had a limited number of physicians who used FMT, making it more difficult to fully comprehend the procedure and its risks and benefits and the attitudes might change if they had a positive experience in treating patients with it.

In conclusion, our study demonstrated a lack of enthusiasm to implement FMT in Jordan by healthcare providers although there is general support for its potential use as a second line of treatment when other traditional medical treatment fails. There are complex ethical, religious, and practice-based challenges that need to be addressed before FMT becomes an established practice. Future studies should examine FMT from local traditional and especially religious perspectives as well as other barriers found in our study, as well as consenting, privacy, and risks. Patient (end-user) perspectives are lacking and would be important to understand the level of acceptability among those who need FMT. Furthermore, there should be providing more education to increase the understanding of FMT benefits and risks among Jordanian healthcare practitioners.

## Data Availability

The data are available upon request from the corresponding author agbakri@ju.edu.jo.

## Abbreviations

FMT: Fecal microbiota transplant
IDSA: Infectious Diseases Society of America
SHEA: Society for Healthcare Epidemiology of America
WSES: World Society of Emergency Surgery
CDI: Clostridium difficile Infection
IRB: Institutional Review Board
DIY: Do-It-Yourself
DTC: Direct-To-Consumer
SD: Standard Deviation
US FDA: The United States Food and Drug Administration
